# Suspected Somatosensory Evoked Potentials Pacing Atria During Cerebral Bypass Surgery: A Case Report

**DOI:** 10.1155/cria/2637445

**Published:** 2025-03-14

**Authors:** Denise S. Abdulahad, Justin Pachuski

**Affiliations:** Department of Anesthesiology and Perioperative Medicine, Penn State Health Milton S. Hershey Medical Center, Hershey, Pennsylvania, USA

**Keywords:** case report, intraoperative neuromonitoring, Moyamoya disease, somatosensory evoked potentials

## Abstract

Somatosensory evoked potentials are commonly utilized during surgery to assess the function of the central nervous system. We present a case of a 48-year-old female patient undergoing cerebral bypass surgery who was noted to have reproducible, stimulation frequency-dependent heart rate changes that coincided with somatosensory evoked potentials stimulation. We hypothesize that the somatosensory evoked potential stimulation current was depolarizing a foci of atrial myocytes, resulting in the initiation of the cardiac cycle.

## 1. Introduction

Moyamoya disease is a progressive cerebrovascular disease that involves the occlusion or stenosis of the carotid arteries with arterial collateral circulation. Patients present with progressive neurological and cognitive deficits. About half of adult patients present with hemorrhage, whereas nearly all pediatric patients present with ischemia [1]. Medical therapy (i.e., aspirin), has shown some benefit in restoring the effect of strokes, but this does not reduce the risk of subsequent strokes. The definitive treatment for Moyamoya disease is surgical management via direct or indirect revascularization, which augments blood flow to the hypo-perfused brain, providing an alternate route of cerebral perfusion [1]. In a direct bypass, a branch of the external carotid artery (usually the superficial temporal artery) is anastomosed to a branch of the internal carotid artery. In contrast, an indirect bypass aims to improve collateral blood flow via angiogenesis [2].

The primary goal of perioperative anesthetic management for cerebral bypass surgery is to balance the cerebral oxygen supply and demand. Maintaining blood pressure between 10% and 20% of the baseline preoperative blood pressure is critical for postoperative neurological outcomes to reduce the risk of ischemic or hemorrhagic stroke [2]. The goal for fluid management is normovolemia, and a hematocrit goal of 30% to 42% has been cited to maintain the balance between blood oxygen-carrying capacity and blood viscosity, as extremes can increase risk of cerebral ischemia [2]. The goal for ventilation is to maintain normocapnia and normoxia. For patients with Moyamoya disease, the areas of the brain supplied by the diseased blood vessels are at higher risk of cerebral ischemia, with both hypercapnia and hypocapnia having negative effects on cerebral perfusion, by either inducing steal phenomenon or cerebral vasoconstriction [2].

Intraoperative neuromonitoring is commonly used to identify intraoperative changes in neural integrity that can guide the surgeon to intervene to decrease the likelihood of neurologic deficits in the postoperative period. Intraoperative neuromonitoring has been widely applied in neurovascular surgeries, including open cranial surgeries, to reduce postoperative neurological deficits [3, 4]. Previous studies have demonstrated its efficacy in reducing instances of postoperative neurologic deficits and in the achievement of better outcomes with open cranial surgeries (e.g., aneurysm clipping) [4]. However, literature has yet to clearly demonstrate that the use of intraoperative neuromonitoring in the setting of superficial temporal artery to middle cerebral artery bypass is associated with improved postoperative outcomes [4, 5]. In our case report, the type of intraoperative neuromonitoring in question was somatosensory evoked potential (SSEP). SSEPs assess the sensory system's integrity, which at the spinal cord level, are primarily contained in the dorsal columns. SSEPs are obtained by electrical stimulation of peripheral nerves: the median or ulnar nerves are commonly stimulated for upper extremity SSEPs, and the posterior tibial nerve at the ankle or the peroneal nerve at the fibular head for capturing lower extremity SSEPs [6].

## 2. Case Description

A 48-year-old-female with Moyamoya disease complicated by multiple ischemic cerebrovascular accidents with mild residual expressive aphasia presented for a superficial temporal artery to middle cerebral artery bypass surgery. Her comorbidities included a spontaneous left anterior descending artery dissection resulting in an anterior wall myocardial infarction which was treated with a drug-eluting stent, and heart failure with preserved ejection fraction.

Induction of anesthesia was unremarkable, and an arterial line was placed for hemodynamic monitoring. General anesthesia was maintained with a total intravenous anesthetic consisting of propofol, ketamine, and remifentanil infusions. Neuromonitoring with SSEPs and motor-evoked potentials were performed throughout the procedure via the Cadwell Cascade PRO IONM System (Kennewick, WA). The lower extremity SSEP stimulation current was applied to the posterior tibial nerves, and upper extremity SSEP stimulation current was applied to the median nerves. The SSEP stimulation current utilized for the posterior tibial nerve was set to 42mAmp at 26 V with an initial repetition rate of 1.96 Hz.

**Figure 1:**
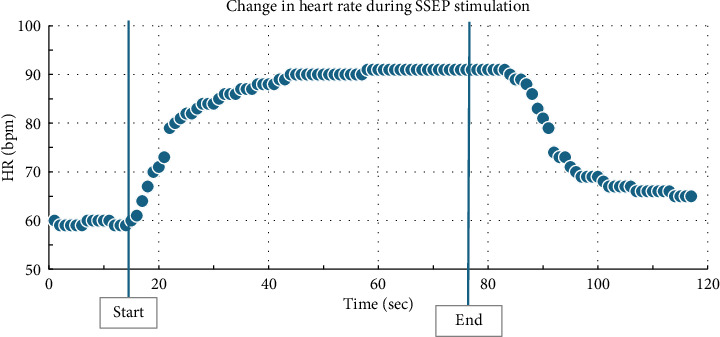
Heart rate changes from start of lower extremity SSEP stimulation at 10 s to the end of SSEP stimulation at 78 s.

**Figure 2:**
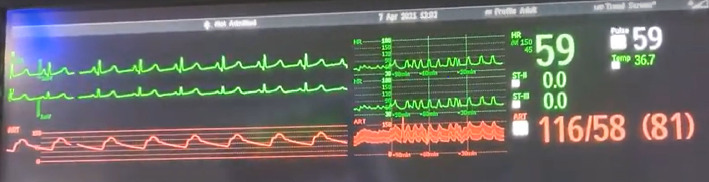
Vitals signs prior to SSEP stimulation.

**Figure 3:**
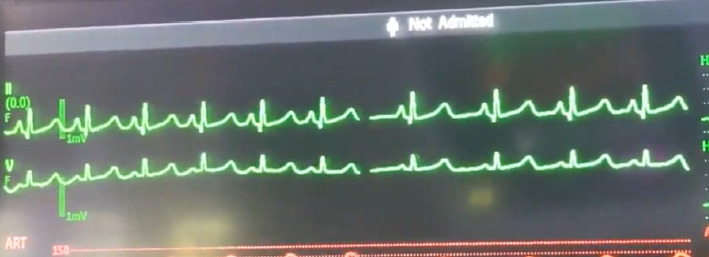
Transition of R-R interval length with SSEP stimulation (left side) and without SSEP stimulation (right side).

**Figure 4:**
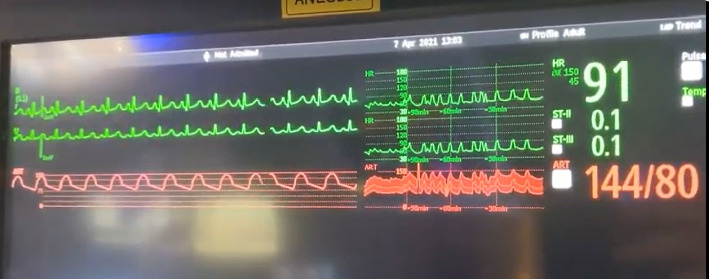
Vitals upon SSEP stimulation with 1.96 Hz, 42 mAmps.

**Figure 5:**
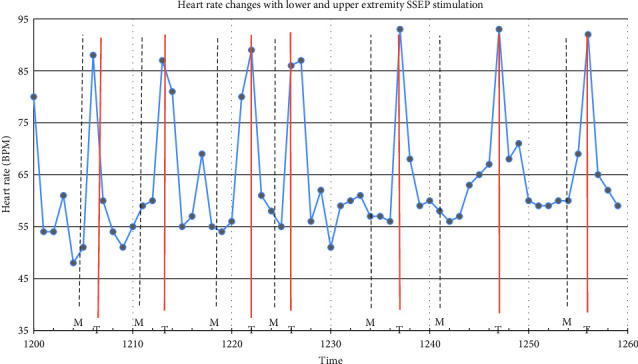
Depiction of heart rate changes over one hour (1200–1259) during SSEP stimulation. The T marks indicate the end of lower extremity SSEP stimulation of the tibial nerves, while the M marks indicate the end of upper extremity SSEP stimulation of the median nerves. There was a marked increase in heart rate with each lower extremity SSEP stimulation, but not with each lower extremity SSEP stimulation.

**Figure 6:**
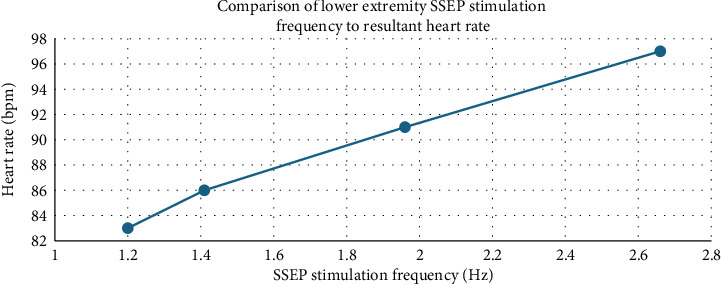
Variable SSEP stimulation frequencies and resultant maximum increase in heart rate.

Intraoperatively, the patient was noted to have labile hemodynamics with brief (∼30 s) episodes of abruptly rising (increasing from 60 beats per minute to 90 beats per minute over a 30 s time period), stable plateau, and then abruptly declining heart rates (decreasing from 90 beats per minute to 60 beats per minute over a 30 s time period) (Figure 1). There were no changes noted in the electrocardiogram morphological waveform during these episodes of relative sinus tachycardia (Figures 2, 3, 4). Initially, this phenomenon was attributed to a light anesthetic depth relative to surgical and neuromonitoring stimulation; however, this was questioned after it was noted that several of these tachycardic episodes occurred during points of very minimal surgical stimulation. Depth of anesthesia remained unchanged during each episode. Upon further investigation and discussion with the neurophysiologist, it was realized that these episodes of reproducible hemodynamic changes coincided with the starting and stopping of SSEP stimulation, specifically during lower extremity SSEP stimulation (posterior tibial nerves), but not upper extremity SSEP stimulation (median nerves) (Figure 5). Normally, heart rate on the Philips IntelliVue X3 monitor is computed by averaging the 12 most recent R-R intervals [7]. While the heart rate changes took about 30 seconds on the monitor, it was noted that immediately after starting and stopping the SSEP stimulation, the R-R interval on the EKG changed (Figure 3). Once the cause of the hemodynamic episodes was suspected, consultation with the neurophysiologist lead to the discovery that the ground lead for the return current was located on the patient's left shoulder. Now that the stimulation source of the lower extremity SSEP was identified as the triggering source, the frequency of stimulation was altered. The SSEP stimulation frequency was directly correlated to the observed increase in heart rate (Figure 6). The heart rate returned to a baseline of about 60 beats per minute after SSEP stimulation was stopped. There was no obvious malfunction of the stimulation device or the electrodes. Other electrical stimulations (motor evoked potentials, train of four) by our neuromonitoring team did not trigger these hemodynamic changes. Neuromonitoring personnel were asked to change the frequency and stimulation of the SSEP to the lowest adequate frequency to minimize the hemodynamic changes associated with the stimulations. SSEP data was able to be provided for the remainder of the procedure by the neuromonitoring team. There were no complications during the case and the patient was successfully extubated and had an unremarkable hospital stay after the procedure.

## 3. Discussion

Electrical current traveling through the heart has been shown to have deleterious effects on the heart rhythm. When cardiac muscle is stimulated by chemical or electrical means, it rapidly depolarizes and the membranes become slightly positive, and this depolarization is represented as the QRS complex [8]. There are various sources of current (i.e., faulty electronic equipment) that may spread through the heart, especially in an operating room environment [8]. A single electric shock may produce an isolated premature ventricular contraction, while strong alternating current may trigger ventricular fibrillation in systole [8]. Arrhythmias related to or coincident with SSEP monitoring have been published in a handful of case reports. In a case series published by Reisener et. al in 2020, the authors hypothesized that SSEP stimulation may trigger a vasovagal reaction that normalizes with the cessation of SSEPs stimulus [9]. A case report published in 1988 by Merritt et al. described implanted pacemaker-induced arrhythmias associated by intraoperative SSEP stimuli during cervical spine surgery. The mechanism of tachycardia in this case was thought to be from the mistaken interpretation by the pacemaker of the SSEP stimulus as its own intrinsic atrial event [10].

Our patient demonstrated a reproducible, stimulation frequency-dependent heart rate change to suggest that the SSEP current was interacting with the conduction system of the heart. Since there was no change in the electrocardiogram QRS morphology, one may infer that the stimulation was acting proximal to the atrioventricular node. The stimulation frequency never exactly matched the resultant heart rate, likely because a certain percentage of those stimulations were reaching the supraventricular region when the myocardium was in the refractory period. Increase in blood pressure also occurred with the increase in heart rate.

We hypothesize that in this case, SSEP stimulation currents were interacting with myocytes in the atria leading to depolarization and increased chronicity of the heart. With each SSEP stimulation initiated by the neurophysiologist, elevations in heart rate and mean arterial pressures from baseline were observed when the posterior tibial nerve was stimulated at various frequencies (current traveling from ankles, though the thorax, then to the ground lead at the left shoulder). No changes were noted when identical currents were applied to the median nerves (where the return current bypassed the heart) (Figure 5). The frequency of stimulation was proportional to the observed changes in heart rate. However, no changes in the electrocardiogram QRS morphology were noted, implying that the currents were interacting with myocytes in the atria leading to depolarization of the ventricles in the standard fashion via the atrioventricular node.

We hypothesize that the patient had a foci of myocytes that were susceptible to depolarization, at or near the sinoatrial node, which were stimulated by SSEP on the posterior tibial nerve. To our knowledge, this phenomena has not been reported before in the literature and should be considered whenever patients have reproducible hemodynamic changes during SSEP stimulation. Care can be taken to alter the ground lead location so that the arc of the stimulation current does not pass through the heart, leading to potential hemodynamic alterations.

With this case report, we hope to bring awareness to the possibility of this phenomena with the goal of altering intraoperative neuromonitoring practices to minimize the likelihood of this occurrence.

## Data Availability

The data that support the findings of this case report are available from the corresponding author upon request.
